# Tracheal stenosis due to cervicothoracic hyperlordosis in patients with cerebral palsy treated with posterior spinal fusion: a report of the first two cases

**DOI:** 10.1186/s12891-021-04094-y

**Published:** 2021-02-23

**Authors:** Yuki Taniguchi, Yoshitaka Matsubayashi, So Kato, Fumihiko Oguchi, Ayato Nohara, Toru Doi, Yasushi Oshima, Sakae Tanaka

**Affiliations:** 1 Department of Orthopaedic Surgery, The University of Tokyo Hospital, 7-3-1 Hongo, Bunkyo-ku, 113-8655 Tokyo, Japan; 2 Department of Next Generation Locomotive Imaging System, The University of Tokyo Hospital, Tokyo, Japan; 3 Department of Spine Surgery, JCHO Tokyo Shinjuku Medical Center, Tokyo, Japan

**Keywords:** Cerebral palsy, Scoliosis, Tracheal stenosis, Spinal deformity, Posterior spinal fusion, Cervicothoracic hyperlordosis, Innominate artery, Case report

## Abstract

**Background:**

Spinal deformity is frequently identified in patients with cerebral palsy (CP). As it progresses, tracheal stenosis often develops due to compression between the innominate artery and anteriorly deviated vertebrae at the apex of the cervicothoracic hyperlordosis. However, the treatment strategy for tracheal stenosis complicated by spinal deformity in patients with CP remains unknown.

**Case presentation:**

This study reports two cases: a 19-year-old girl (case 1) and a 17-year-old girl (case 2), both with CP at Gross Motor Function Classification System V. Both patients experienced acute oxygen desaturation twice within the past year of their first visit to our department. X-ray and computed tomography revealed severe scoliosis and cervicothoracic hyperlordosis causing tracheal stenosis at T2 in case 1 and at T3-T4 in case 2, suggesting that their acute oxygen desaturation had been caused by impaired airway clearance due to tracheal stenosis. After preoperative halo traction for three weeks, both patients underwent posterior spinal fusion from C7 to L5 with Ponte osteotomy and sublaminar taping at the proximal thoracic region to correct cervicothoracic hyperlordosis and thoracolumbar scoliosis simultaneously. Postoperative X-ray and computed tomography revealed that the tracheal stenosis improved in parallel with the correction of cervicothoracic hyperlordosis. Case 1 did not develop respiratory failure 1.5 years after surgery. Case 2 required gastrostomy postoperatively due to severe aspiration pneumonia. However, she developed no respiratory failure related to impaired airway clearance at one-year follow-up.

**Conclusions:**

We present the first two cases of CP that developed tracheal stenosis caused by cervicothoracic hyperlordosis concomitant with progressive scoliosis and were successfully treated by posterior spinal fusion from C7 to L5. This enabled us to relieve tracheal stenosis and correct the spinal deformity at the same time. Surgeons must be aware of the possibility of coexisting tracheal stenosis in treating spinal deformity in patients with neurological impairment because the surgical strategy can vary in the presence of tracheal stenosis. This study demonstrated that some patients with CP with acquired tracheal stenosis can be treated with spinal surgery.

## Background

Spinal deformity is frequently identified in individuals with cerebral palsy (CP) [1–3]. The incidence of scoliosis is associated with the severity of CP and in patients at the lowest level of Gross Motor Function Classification System (GMFCS) V, scoliosis with a Cobb angle ≥ 40° was reported to be seen in 75 % of the individuals at the age of 20 years [3]. Moreover, the spinal curve in these patients has a progressive nature due to increased muscle tone, and especially when the magnitude of the curve exceeds 40° before the age of 15 years, continuous progression is expected even after the end of growth [4]. As spinal deformity progresses, tracheal stenosis often develops caused by compression between the innominate artery and the anteriorly deviated vertebrae at the apex of the cervicothoracic hyperlordosis and can subsequently lead to acute fatal respiratory failure because of impaired airway clearance[5–8]. However, the treatment strategy for tracheal stenosis complicated by spinal deformity in these patients has not yet been determined, and no study has described such cases treated with spinal surgery. Herein, we report the first two cases of CP that developed tracheal stenosis caused by cervicothoracic hyperlordosis concomitant with progressive scoliosis and were successfully treated with posterior spinal fusion (PSF) from C7 to L5, enabling us to relieve tracheal stenosis and correct the spinal deformity at the same time.

## Case presentation

### Case 1

**Fig. 1:**
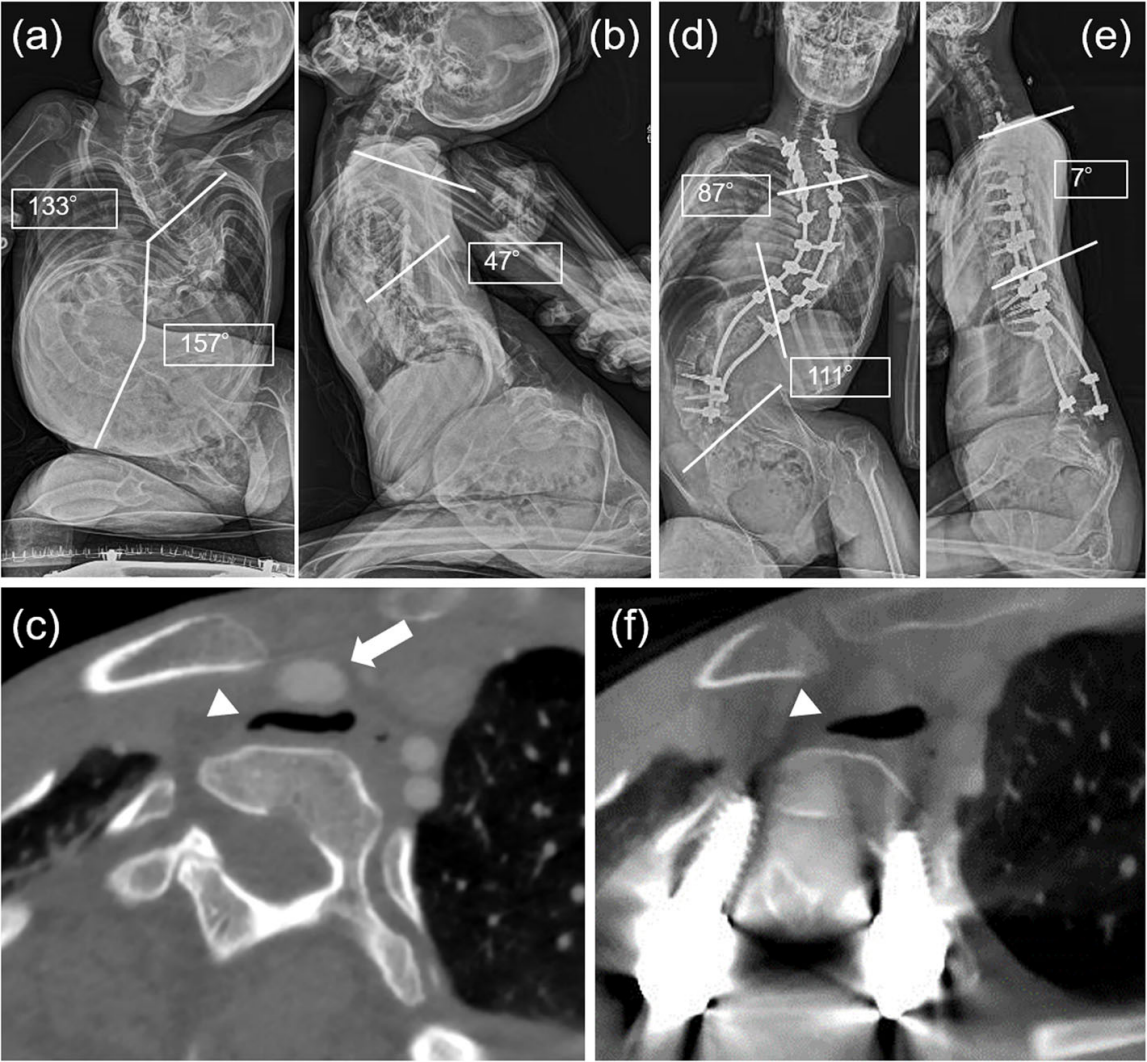
Image findings of case 1. **a**, **b** Preoperative posterior-anterior and lateral X-rays of the whole spine in assisted sitting position shows severe scoliosis with exaggerated cervicothoracic hyperlordosis. **c** Preoperative contrast enhanced computed tomography at T2 demonstrates a flattened tracheal lumen between innominate artery and anteriorly deviated vertebra. **d**, **e** Postoperative posterior-anterior and lateral X-rays of the whole spine in assisted sitting position reveals well-corrected cervicothoracic alignment. **f** Postoperative computed tomography demonstrates the expansion of the narrowed tracheal lumen.The white arrow in (**c**) indicates the innominate artery compressing the trachea from the anterior aspect. The white arrowheads in (**c**) and (**f**) indicate the tracheal lumen

A 19-year-old girl with CP at GMFCS V was referred to our department for treatment of a progressive spinal deformity. She had no history of respiratory failure until the age of 14 years. Within the past year of her first visit to our department, she was ambulanced to the ER twice due to acute oxygen desaturation. X-ray in assisted sitting position and computed tomography (CT) demonstrated severe scoliosis with a Cobb angle of 157 ° and tracheal stenosis at T2 due to compression between the innominate artery and the cervicothoracic hyperlordosis, suggesting that her acute oxygen desaturation had been caused by the impaired airway clearance at this level (Fig. 1a-c). After preoperative halo traction for three weeks, she underwent PSF from C7 to L5 with Ponte osteotomy at T1/2/3/4 and sublaminar taping from T1 to T6 to correct cervicothoracic hyperlordosis and thoracolumbar scoliosis simultaneously. Postoperative X-ray and CT revealed that her tracheal stenosis improved in parallel with the correction of cervicothoracic hyperlordosis. She had not developed respiratory failure until 1.5 years after surgery (Fig. 1d-f).

### Case 2

**Fig. 2:**
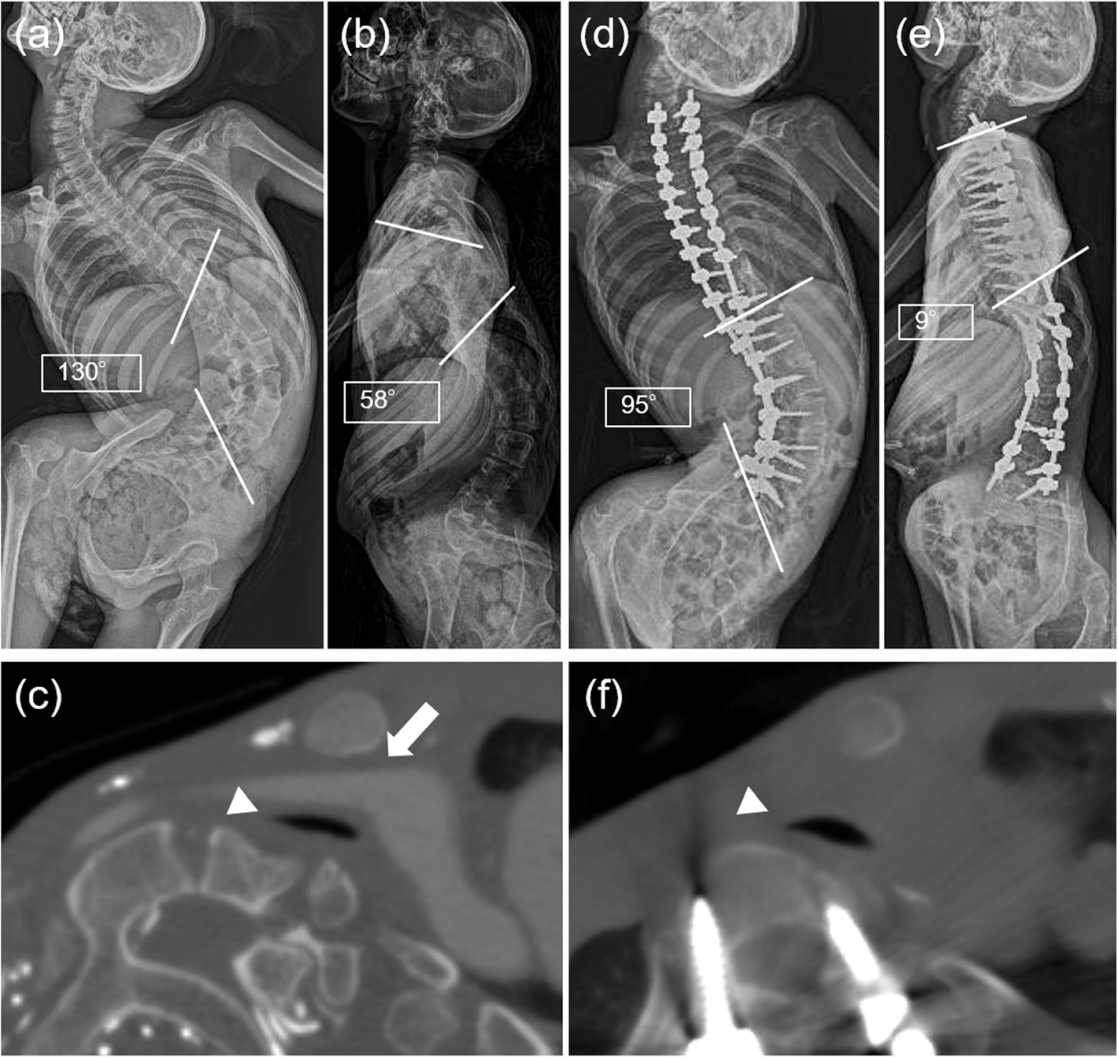
Image findings of case 2. **a**, **b** Preoperative anterior-posterior and lateral X-rays of the whole spine in supine position shows severe scoliosis with exaggerated cervicothoracic hyperlordosis. **c** Preoperative contrast enhanced computed tomography at T3/4 level demonstrates flattened tracheal lumen between innominate artery and anteriorly deviated vertebra. **d**, **e** Postoperative anterior-posterior and lateral X-rays of the whole spine in supine position reveals well-corrected cervicothoracic alignment. **f** Postoperative computed tomography demonstrates the improvement of tracheal stenosis. The white arrow in (**c)** indicates the innominate artery compressing the trachea from the anterior aspect. The white arrowheads in (**c**) and (**f**) indicate the tracheal lumen

A 17-year-old girl with CP at GMFCS V was referred to our department for surgical treatment of her spinal deformity. Within the past year of her first visit to our department, she needed emergency hospitalization twice for acute respiratory failure and has been recommended for tracheostomy due to tracheal stenosis. Although her preoperative anterior-posterior radiograph in the supine position showed thoracolumbar scoliosis with a Cobb angle of 130 °, which seemed to have little impact on her thoracic cavity, preoperative lateral X-ray and CT clearly demonstrated a flattened tracheal lumen at the T3/4 level due to exaggerated cervicothoracic hyperlordosis (Fig. 2a-c). After preoperative halo traction for three weeks, she underwent PSF from C7 to L5 with Ponte osteotomy at T2/3/4/5/6 and sublaminar taping from T2 to T5 like case 1 (Fig. 2d, e). Postoperative CT revealed expansion of the tracheal lumen (Fig. 2f). Although she required gastrostomy postoperatively due to severe aspiration pneumoniae, she developed no respiratory failure related to impaired airway clearance during the one-year follow-up period without having a tracheostomy.

## Discussion and conclusions

Children with severe CP often develop acquired airway obstruction and sometimes need tracheostomy. However, the frequency or etiology of this condition in these patients has not yet been fully elucidated [9, 10]. Few reports have described neurologically impaired patients who developed tracheal stenosis at the thoracic inlet level pinched between the innominate artery and the anteriorly deviated vertebrae [5–8, 11]. (Table 1) Based on the findings of earlier reports, we hypothesized that progressive anterior deviation of the vertebrae at this level, which occurs concomitantly with the progression of spinal deformity, can be a critical cause for developing tracheal stenosis in patients with CP [5–8, 11].

**Table 1:** Previous reports of the neurologically impaired patients developing tracheal stenosis between innominate artery and spine

Authors (Year) [Reference]	Number of cases	Mean patient age in years (range)	Mean f/u in months (range)	Treatment	Postoperative course
Tatekawa Y. (2011) [7]	6	12 (9–15)	24 (8–45)	Superior mediastinal exposure, external reinforcement with autologous cartilage graft, anterior sling of the innominate artery with a muscle sling, and tracheopexy	Recurrence in one patient
Grillo HC (2005) [6]	1	28	108	Partial upper sternotomy	No recurrence
Tsugawa C (2004) [8]	2	5 (4–6)	20 (4–36)	Transection of the innominate artery	No recurrence
Tanaka M (2001) [5]	3	26 (18–35)	^a^6	Tracheal stent in one patient	^a^Died 6 months postoperatively
Obatake M (2011) [11]	1	11	unknown	Transection of the innominate artery	unknown
Taniguchi Y (2021) [this study]	2	18 (17–19)	15 (12–18)	Posterior spinal fusion	No recurrence

*f/u* follow-up

^a^Regarding the patient who underwent a tracheal stent

There has been no established treatment strategy for tracheal stenosis in patients with CP. Tracheostomy cannot be the gold standard because tracheostomy for a narrowed tracheal wall has a potential risk for fatal trachea-innominate artery fistula [12–14]. Transection of the innominate artery is also option for tracheal stenosis in patients with CP, both with and without tracheostomy. However, the transection of the innominate artery can be accompanied by ischemia of the right upper extremity [11, 15]. Tatekawa et al. reported a surgical strategy for acquired tracheomalacia due to innominate artery compression of the trachea in six patients with permanent neurological impairment [7]. The surgical procedure included mediastinal exposure, external reinforcement with autologous cartilage graft, anterior sling of the innominate artery with muscle sling, and tracheopexy. However, tracheomalacia recurred in one patient due to mucosal infolding secondary to the deformed spine [7]. Their recurrent case convinced us of the importance of radical treatment for the spinal deformity causing tracheal stenosis, as we did for the two patients in the present study.

Regarding the spinal deformity, both patients presented with cervicothoracic hyperlordosis, which caused anterior deviation of the vertebrae at the level of the thoracic inlet and compressed the trachea from the posterior aspect. Although it is obvious that cervicothoracic hyperlordosis is a critical factor for tracheal stenosis in the present two cases, the surgical strategy for this condition has not yet been determined. In our two cases, we adopted the same surgical strategy, comprising the selection of the upper instrumented vertebra (UIV) at C7 and multiple sublaminar taping with Ponte osteotomy in the proximal thoracic region. Ponte osteotomy, also known as posterior column osteotomy, which involves the resection of posterior bony elements, including the entire facet complexes and ligamentum flavum, carries the potential risk of destabilizing the spine, especially at the cervicothoracic junction. We applied this procedure in this region because correction of hyperlordosis at this site was mandatory for these patients and Ponte osteotomy is reported to be associated with better kyphosis restoration [16–18]. Furthermore, in addition to using pedicle screws, we utilized multiple sublaminar taping with Ponte osteotomy to pull up the ventrally deviated vertebra. In general, pedicle screws alone are not suitable for pulling up the vertebra, especially in patients with osteoporosis such as CP. The selection of UIV in these patients may require some discussion. Regarding the selection of UIV, because the apex level of the cervicothoracic hyperlordosis was T2 in case 1 and T3/4 in case 2, setting UIV at least in the cervical region rather than at T2 was indicated, which is usually selected as UIV in neuromuscular scoliosis. We selected UIV at C7 in both patients because securing strong anchors, such as pedicle screws or hooks, would be difficult above C7 due to anatomical features; however, the adequate UIV in these cases remains to be elucidated, considering the huge physiological stress at the cervicothoracic junction. It is another matter of debate whether the thoracolumbar curve in addition to the main thoracic curve should be involved in the fusion area in these patients because extensive spinal fusion may lead to prolonged operation time and can be associated with a higher incidence of complications. We decided to involve a thoracolumbar curve in the fusion area because the thoracolumbar curve can also influence the respiratory status, probably due to compression of the diaphragm [19]. Our surgical strategy was actually effective for relieving tracheal stenosis in the present two cases; however, whether our surgical strategy can always be applied for all patients with the same condition is not yet established.

The frequency or predictive factors for developing cervicothoracic hyperlordosis in patients with neurological impairment are still unknown. Therefore, it is difficult to predict the development of tracheal stenosis occurring concomitantly with the progression of spinal deformity. Hence, surgeons must be aware of the possibility of coexisting tracheal stenosis in treating spinal deformity in these patients and check the condition of the thoracic vertebral bodies and trachea on CT preoperatively, which is the most suitable modality for depicting these anatomical structures, as surgical strategy can vary in the presence of tracheal stenosis.

There are some limitations to this case report. First, due to the small number of cases in this study, it was difficult to conclude the definite effectiveness of our surgical strategy in patients with the same condition. Second, because of the short-term follow-up period, the long-term outcomes of these patients remain unknown. Third, it is yet to be elucidated which treatment option for tracheal stenosis shown in (Table 1) is most suitable for each patient.

In conclusion, we present the first two cases of CP that developed tracheal stenosis caused by cervicothoracic hyperlordosis concomitant with progressive scoliosis and were successfully treated with PSF, although the follow-up period was limited. This study demonstrated that some patients with CP with acquired tracheal stenosis can be treated with spinal surgery. Although our surgical strategy was effective for the present two cases, further investigation is needed to clarify whether it can always be applied for all patients with the same condition.

## Data Availability

The datasets used and/or analyzed during the current study are available from the corresponding author upon reasonable request.

## Abbreviations

CP: Cerebral palsy
GMFCS: Gross Motor Function Classification System
PSF: Posterior spinal fusion
CT: Computed tomography
UIV: Upper instrumented vertebra
